# Case of Punctured Wound of the Brain—Recovery

**Published:** 1871-05

**Authors:** C. T. Fenn

**Affiliations:** Chicago


### ARTICLE VI.—*Case of Punctured Wound of the Brain—Recovery.* By C. T. FENN, M.D., Chicago.

S. M. G., a robust boy, aged three and a half years, fell from the top of a fence three and a half feet, striking his head on a rusty nail fixed in a post that lay on the ground. His cries brought relief. It was found that the head and free portion of the nail, one inch in length, was driven directly through the skull near the posterior superior angle of the left parietal bone. The child's head was fixed to the post. A half hour later the puncture was probed to the depth of one inch, and hair and spiculæ of bone were extracted. There was little evidence of shock. A light cold wet compress was applied and kept in position for one week. This was the only treatment. No morbid symptom followed the injury. The child played, ate, and slept, with as great vivacity as before. The wound soon presented granular edges, but remained open and discharged freely for several days. Sept. 29th, nine days after the accident, he had a convulsion. The sole cause appeared to be a slight diarrhœa, with slimy stools. A few doses of Dovers powder were given to check the diarrhœa. After this nothing occurred. The wound was entirely healed in twenty-two days—the surface having been carefully kept open to the last.

It is to be observed that the effect of concussion was comparatively slight, modified, perhaps, by the perforation of the skull. The results of inflammation of the brain were certainly averted by the free opening which was maintained.

